# Right Colotomy for Chronic Colitis

**Published:** 1899-05-27

**Authors:** 


### RIGHT COLOTOMY FOR CHRONIC COLITIS.

"The colon is not an organ which is necessary for the preservation of perfect health." This gives the clue to the treatment of some of the most obstinate and distressing diseases of the lower bowel, for by the performance of a right colotomy the whole of the bowel below the incision can be set at rest, and left to heal. The introduction of this method of treating the more severe degrees of colitis is a most important advance, for the conditions referred to are not only most tedious and distressing, but, if not relieved, are all too apt to have a fatal issue. At the last meeting of the Clinical Society, Dr. Hale White and Mr. Golding Bird contributed a paper giving details of three additional cases in which this treatment has been adopted with most satisfactory results. They recommend palliative or curative right-sided colotomy for severe and otherwise hopeless examples of the following diseases: (1) Intractable membranous colitis; (2) all forms of chronic ulceration of the colon that have resisted medical treatment and are obviously otherwise incurable, but they consider that most cases of very chronic dysentery are to be cured without colotomy; and (3) cases of idiopathic dilatation of the colon. Colotomy seems to be preferable to cæcotomy, for when the latter operation has been performed fluid fæces have escaped from the artificial anus, and this has given rise to much trouble; moreover, it was difficult to prevent some fæces passing on into the colon. It does not seem to be necessary to wash out the bowel from the artificial to the natural anus, and the authors consider that, although more experience is necessary before deciding upon the time for which the artificial anus should be left open, this should certainly not be less than six months.

